# MGUS prevalence among active-duty military personnel

**DOI:** 10.1038/s41408-026-01511-0

**Published:** 2026-05-07

**Authors:** Dickran Kazandjian, Christin DeStefano, Sarah Darmon, Alexander Dew, Elizabeth Hill, Katie Thoren, Ola Landgren

**Affiliations:** 1 Sylvester Cancer Center, University of Miami, Miami, FL USA; 2 Walter Reed National Military Medical Center, Bethesda, MD USA; 3 Murtha Cancer Center Research Program, Department of Surgery, Uniformed Services University of the Health Sciences, Bethesda, MD USA; 4 The Henry M. Jackson Foundation for the Advancement of Military Medicine, Inc., Bethesda, MD USA; 5 Duke University Health Care System, Durham, NC USA; 6 National Cancer Institute, National Institutes of Health, Bethesda, MD USA

**Keywords:** Risk factors, Myeloma

## Abstract

Monoclonal gammopathy of undetermined significance (MGUS) is a precursor condition for multiple myeloma, and although environmental exposures have been implicated in its pathogenesis, it is unknown whether exposure to open-air burn pits used during U.S. military operations in Iraq affects prevalence of MGUS in active-duty service members (ADSMs). ADSMs represent a distinct young and physically fit population, with higher proportions of male and Black individuals compared to the general U.S. population. This retrospective cohort study evaluated MGUS prevalence among ADSMs. Participants included ADSMs deployed to Iraq with self-reported burn pit exposure (DEP-IQ; *n* = 534), ADSMs deployed to Germany without burn pit exposure (DEP-DEU; *n* = 534), and non-deployed ADSMs (NON-DEP; *n* = 521). Eligibility criteria included deployment ≥180 days, age ≥35 years at deployment, and ≥10 years of continued military service. Serum samples collected 11–14 years post-deployment were analyzed using serum protein electrophoresis, immunofixation, and serum free light chain (sFLC) assays. MGUS and light-chain MGUS (LC-MGUS) prevalence were estimated using conventional and revised age-stratified sFLC reference intervals. MGUS + revised LC-MGUS prevalence did not differ between DEP-IQ (5.6%), DEP-DEU (4.3%), and NON-DEP (5.2%) cohorts. The overall total prevalence of MGUS + revised LC-MGUS was 5% and was higher in Black (10.4%) than White ADSMs (3.9%), *p* < 0.001. When using conventional sFLC reference intervals, LC-MGUS was more frequently identified among deployed ADSMs (1.6%) compared with non-deployed ADSMs (0.2%); however, this association was no longer significant when revised reference intervals were applied. These findings suggest that military service may involve cumulative or non–burn pit exposures contributing to plasma cell dysregulation. Application of updated sFLC reference ranges is critical to avoid overestimation of LC-MGUS. Long-term studies are warranted to define progression risk and inform targeted screening strategies in ADSMs.

## Background

Monoclonal gammopathy of undetermined significance (MGUS) is a common, asymptomatic plasma-cell proliferative disorder that has been identified as a precursor to multiple myeloma (MM) and other related hematologic malignancies and diseases. Although MGUS itself is benign, virtually all cases of MM are preceded by MGUS, necessitating lifelong clinical surveillance following diagnosis [1, 2]. MGUS is defined by the presence of <10% clonal plasma cells in the bone marrow and a detectable monoclonal immunoglobulin (M protein) or free *κ* or *λ* light chains in the serum, classified as light-chain (LC) MGUS. These conditions are identified through peripheral blood testing, using serum protein electrophoresis (SPEP) or immunofixation electrophoresis (IFE) for the detection of intact monoclonal immunoglobulins and with serum free light chain (sFLC) measurements for isolated light-chain abnormalities [3]. MGUS progresses to multiple myeloma at an estimated annual rate of approximately 1%; however, individual risk varies considerably and is influenced by several disease-related factors, such as the level and isotype of the monoclonal protein, abnormalities in the serum free light-chain ratio, the extent of bone marrow plasma cell involvement, and the proportion of clonally restricted plasma cells [4, 5].

The pathogenesis of MGUS, as well as the factors that contribute to its progression into more proliferative or malignant hematologic disorders, is not fully understood. However, epidemiologic and population-based cohort studies indicate that MGUS is relatively common among older adults and disproportionately affects certain racial groups, with an estimated prevalence of approximately 3–5% among individuals aged ≥50 years and a roughly twofold higher prevalence among individuals of African descent [6–9]. Further supporting this substantial body of evidence, one of the largest population-based screening studies of monoclonal gammopathies to date reported that, among approximately 75,000 individuals aged ≥40 years living in Iceland, MGUS was identified in 2.3% of individuals aged 40–59 years and in 3% across the entire study population (ages 40–104) [10]. Outside of these factors, accumulating evidence suggests a role for environmental and occupational exposures in MGUS development and progression. Epidemiologic studies have also reported associations between MGUS, MM, and occupational exposures from agricultural chemicals, industrial solvents, and other workplace-related agents. For example, pesticide exposure has been associated with an increased incidence of MGUS, implicating dioxins and related compounds [11, 12]. Similarly, the World Trade Center (WTC) attacks on September 11, 2001, exposed first responders to aerosolized dust and gases containing known carcinogens, including polychlorinated biphenyls (PCBs) and polycyclic aromatic hydrocarbons (PAHs). A screening study of firefighters who served as first responders at the WTC demonstrated a significantly higher age-standardized prevalence of MGUS and combined MGUS/LC-MGUS compared with reference populations [13].

Within this context, U.S. active-duty service members (ADSMs) represent a distinct population with characteristics that may influence MGUS risk [14]. ADSMs include a higher proportion of men and Black individuals compared with the general U.S. population [15]. Although typically young, fit, and healthy, maintaining strict physical fitness standards, undergoing regular mental health evaluations, and having uniform access to comprehensive healthcare, the advantages of this “healthy soldier effect” may be counterbalanced by service in austere environments, including military deployments and potential toxic exposures [16]. Deployment-related exposures among U.S. military personnel warrant particular consideration, given the unique intensity, duration, and complexity of environmental and chemical exposures encountered during military service. For example, during the Vietnam War, millions of service members were exposed to Agent Orange, a defoliant contaminated with the highly toxic dioxin 2,3,7,8-tetrachlorodibenzo-p-dioxin (TCDD). One notable study showed that in a cohort of Vietnam Era Veterans, high-level exposure to Agent Orange was linked to a 48% increased risk of progression into MM in individuals with MGUS [17]. These observations underscore the relevance of deployment-related toxicant exposures in shaping MGUS risk and disease course among military personnel. Importantly, herbicide exposure during the Vietnam War represents only one historical example of sustained chemical exposure during military service, with more recent conflicts introducing distinct but similarly complex exposure profiles.

Open air burn pits were extensively used during U.S. military operations in Iraq and Afghanistan to dispose of waste, including plastics, electronics, chemicals, and medical materials [18, 19]. These pits emitted a complex mixture of toxic airborne substances, raising concerns about their long-term impact on health [20]. Characterization studies have identified numerous hazardous components in burn pit emissions, including fine particulate matter, volatile organic compounds, heavy metals, and notably, compounds with established roles in hematologic carcinogenesis, which have been implicated in MGUS and MM, including particulate matter, dioxins, and polycyclic aromatic hydrocarbons [18, 19]. Despite this biological plausibility, epidemiologic evidence linking burn pit exposure or deployment-related environmental exposures during U.S. military operations in Iraq and Afghanistan to subsequent development of hematologic malignancies remains limited and inconclusive. Retrospective cohort studies evaluating cancer risk among burn pit-exposed ADSMs have been sparse, and several investigations have reported no significant increase in overall cancer incidence or hematologic malignancies among exposed service members compared with matched controls [15, 21]. Although existing research on deployment-related exposures and hematologic malignancy risk has produced largely inconclusive findings, the demographic profile of individuals who typically develop these malignancies, together with the carcinogenic properties of burn pit emissions, underscore the need for continued longitudinal follow-up of service members who participated in the Iraq and Afghanistan conflicts. Accordingly, evaluating early plasma cell precursor conditions such as MGUS may provide a more sensitive approach for detecting potential long-term hematologic effects of deployment-related exposures than studies limited to overt malignancy endpoints, which can take decades to occur.

Interpretation of MGUS and LC-MGUS prevalence in military cohorts is further complicated by methodological considerations, particularly with respect to LC-MGUS classification. Recent studies have suggested that commonly used reference intervals for sFLCs may be inaccurate; specifically, standard reference ranges for *κ* FLC, *λ* FLC, and the FLC ratio in individuals with preserved kidney function have been shown to result in overdiagnosis of *κ* light-chain and underdiagnosis of *λ* light-chain monoclonal gammopathies [22]. In a recent analysis of data collected from the Iceland Screens, Treats, or Prevents Multiple Myeloma (iStopMM) study, Long et al. proposed revised, age-stratified reference intervals for serum *κ* FLC, *λ* FLC, and the FLC ratio, along with an updated definition of LC-MGUS [10, 22]. Given the relatively young age, preserved renal function, and potential for exposure-related immune perturbations in active-duty military populations, application of these updated reference intervals is particularly relevant to avoid misclassification and to ensure accurate estimation of LC-MGUS prevalence in this cohort.

Accordingly, we conducted a retrospective cohort study to evaluate the prevalence of monoclonal MGUS and LC-MGUS among ADSMs deployed to the Iraqi military installations Joint Base Balad and Camp Taji between January 1, 2005, and June 30, 2007, compared with ADSMs deployed to Germany and non-deployed ADSMs serving as control groups. We hypothesized that deployment to Iraq would be associated with a higher prevalence of MGUS and LC-MGUS compared with internationally deployed and non-deployed ADSMs, and that reported burn pit exposure would confer additional risk beyond deployment alone. Therefore, the objectives of this study were to: (1) estimate the prevalence of MGUS and LC-MGUS among ADSMs deployed to these locations relative to deployed and non-deployed control groups; (2) assess the association between international deployment and MGUS/LC-MGUS prevalence; and (3) evaluate whether burn pit exposure independently contributes to MGUS and LC-MGUS risk.

## Methods

This study was acknowledged by the institutional review boards (IRB) and required regulatory authorities, including the University of Miami and the Uniformed Services University of the Health Sciences Human Subjects Protection Office.

### Study population and data sources

Serum samples and clinical data (*N* = 1589) were attained from the Armed Forces Health Surveillance Division (AFHSD) epidemiologic health registry and biorepository after IRB exemptions. All serum samples and clinical data had been obtained from individuals who served in the U.S. Armed Forces, either in the Army or the Air Force.

Samples and data were obtained across three different cohorts: (1) the exposed cohort, ADSMs who deployed to Iraq and reported burn pit exposure (DEP-IQ, *N* = 534), (2) ADSMs who were deployed to Germany (DEP-DEU, *N* = 534), and (3) ADSMs who never deployed (NON-DEP, *N* = 521). The DEP-DEU and NON-DEP cohorts served as control groups to minimize confounding related to overseas service and the healthy deployer effect. For non-deployed ADSMs, samples included were collected within (±) 2 years of the DEP-IQ cases.

### Cohort definitions and eligibility criteria

#### Deployed cohorts: DEP-IQ, exposed, and DEP-DEU, control

Serum samples were eligible for inclusion in the deployed cohorts (DEP-IQ and DEP-DEU) if they were obtained from service members who: (1) deployed for ≥ 180 days, (2) were ≥ 35 years old at time of deployment, (3) remained on active duty ≥ 10 years, and (4) had post-deployment serum samples available in the AFHSD. Deployment-related characteristics were documented for the DEP-IQ and DEP-DEU cohorts, including deployment location and duration of the qualifying deployment. Samples for deployed cohort (DEP-IQ and DEP-DEU) were collected a median of approximately 11 years (range ~10–14 years) after deployment.

#### DEP-IQ

Cases that were eligible for the DEP-IQ cohort were (1) U.S. service members who deployed to Joint Base Balad or Camp Taji in Iraq between January 1, 2005, and June 30, 2007, and (2) who had self-reported exposure to burn pit smoke, burning trash, or similar airborne hazards as documented on a post-deployment health assessment. Camp Taji is located in a rural region approximately 27 km north of Baghdad, Iraq. Joint Base Balad is located near Balad, Iraq, approximately 64 km north of Baghdad.

#### DEP-DEU, control cohort

Cases that were eligible for the DEP-DEU cohort were (1) U.S. service members who deployed to Germany, (2) had never been deployed to Southwest Asia nor had burn pit exposure, and (3) did not self-report exposure to burn pit smoke, burning trash, or similar airborne hazards.

#### Non-deployed cohort: NON-DEP, control

Cases that were eligible for the NON-DEP cohort were (1) U.S. service members who never deployed, (2) were actively serving during the same period as the DEP-IQ and DEP-DEU cohorts, and (3) had specimens collected within ±2 years of DEP-IQ cases. Samples for NON-DEP were collected a median of approximately 11 years post their DEP-IQ matched time of service (range ~10–15 years).

### Demographic and military service characteristics

Demographic and military service characteristics were obtained for all three cohorts, including age, deployment duration in days, race, sex, military occupation during service, rank, and service branch (Table 1). The DEP-IQ cohort was matched 1:1 to the DEP-DEU and NON-DEP cohorts using deployment year (±10 years), age (±3 years), sex, service branch, rank, and occupational category.

**Table 1:** Characteristics of ADSMs deployed to Iraq (Burn Pit Exposed Cohort (DEP-IQ), Germany (Deployed Control Cohort (DEP-DEU)) and non-deployed (NON-DEP)).

	No (%)			
Characteristic	All ADSMs (*N* = 1589)	DEP-IQ	DEP-DEU	NON-DEP
		(*N* = 534)	(*N* = 534)	(*N* = 521)
**Median age at deployment, years (range)**	37(31–55)	37(35–52)	37(35–55)	37(31–54)
**Deployment duration**, median days	320	243	852	N/A
**Race**				
White	1033(65.0%)	346(64.8%)	346(64.8%)	341(65.5%)
Black	298(18.8%)	100(18.7%)	100(18.7%)	98(18.8%)
Hispanic	118(7.4%)	40(7.5%)	40(7.5%)	38(7.3%)
Other	140(8.8%)	48(9.0%)	48(9.0%)	44(8.4%)
**Male sex**				
**Occupation**	1415(89%)	475(89%)	475(89%)	465(89.3%)
Repair/Engineering	434(27.3%)	147(27.5%)	147(27.5%)	140(26.9%)
Communications/Intelligence	355(22.3%)	119(22.3%)	119(22.3%)	117(22.5%)
Infantry/Artillery/Combat engineering	246(15.5%)	82(15.4%)	82(15.4%)	82(15.7%)
Healthcare	156(9.8%)	52(9.7%)	52(9.7%)	52(10.0%)
Pilot/Aircrew	72(4.5%)	24(4.5%)	24(4.5%)	24(4.6%)
Motor transport	67(4.2%)	23(4.3%)	23(4.3%)	21(4.0%)
Other	259(16.3%)	87(16.3%)	87(16.3%)	85(16.3%)
**Rank**				
Enlisted	995(62.6%)	335(62.7%)	335(62.7%)	325(62.4%)
Officer	594(37.4%)	199(37.3%)	199(37.3%)	196(37.6%)
**US Military Service Branch**				
Army	1513(95.2%)	508(95.1%)	508(95.1%)	497(95.4%)
Air Force	76(4.8%)	26(4.9%)	26(4.9%)	24(4.6%)

Baseline demographics for each cohort and the combined group.

### Serum specimen analysis

Serum samples from deployed (DEP-IQ and DEP-DEU) and non-deployed (NON-DEP) cohorts were analyzed for monoclonal proteins by immunofixation electrophoresis (IFE) using pentavalent antisera. IFE-positive samples were confirmed and immunotyped using IFE gels (Sebia). SFLC concentrations were measured using a commercial assay (Sebia) on the DYNEX Agility platform. Samples with detectable monoclonal proteins by IFE underwent serum protein electrophoresis (SPEP) quantification by capillary electrophoresis (Sebia).

### Statistical analysis

Descriptive statistics were used to summarize demographic, military service, and deployment characteristics across cohorts. Continuous variables were summarized using medians and ranges, and categorical variables were summarized as counts and percentages. Differences in baseline characteristics between cohorts were assessed using the Fisher's exact test.

MGUS was evaluated by the detection of an M-protein detected on SPEP or IFE. LC-MGUS was evaluated using both conventional and revised, age-stratified reference intervals utilizing data derived from the iStopMM study, as described by Long et al. [22]. The conventional definition of LC-MGUS was a FLC ratio outside the reference interval with an increase in the involved *κ* or *λ* FLC, without evidence of M protein on SPEP or IFE. Because all ADSMs included were < 70 years old and with presumed normal renal function, the revised definition of LC-MGUS was a FLC ratio <0.44 or >2.16, with *κ* > 39.0 mg/L or *λ* > 36.7 mg/L. Prevalence estimates were calculated with corresponding 95% confidence intervals (CIs). Comparisons of MGUS and LC-MGUS prevalence between deployed and non-deployed cohorts, as well as between burn pit-exposed and unexposed service members, were performed using *χ*² tests.

## Results

### Study population

A total of 1589 ADSMs were included in the analysis, comprising 534 DEP-IQ, 534 DEP-DEU, and 521 NON-DEP. Demographic and military service characteristics are summarized in Table 1. The median age at deployment or matched reference time was 37 years (range, 31–55) and was similar across cohorts. Overall, 89% of participants were male, and the racial composition was 65.0% White, 18.8% Black, 7.4% Hispanic, and 8.8% other racial groups, with comparable distributions across cohorts. Military occupation, rank, and service branch were well balanced, reflecting successful matching. Among deployed cohorts, median deployment duration differed by location, with 243 days for DEP-IQ and 852 days for DEP-DEU.

### Prevalence of MGUS and LC- MGUS across cohorts

The overall prevalence of MGUS, defined by an immunofixation-positive monoclonal protein, was 4.7% (95% CI, 3.7–5.9 per 100 persons) (Table 2). MGUS prevalence estimates were similar across groups: 5.1% (27/534) in the DEP-IQ cohort (95% CI, 3.4–7.3 per 100 persons), 3.9% (21/534) in the DEP-DEU cohort (95% CI, 2.5–5.9 per 100 persons), and 5.2% (27/521) in the NON-DEP cohort (95% CI, 3.4–7.5 per 100 persons). Group comparisons did not identify statistically significant differences between the DEP-IQ and DEP-DEU cohorts (*p* = 0.23) or between the DEP-IQ and NON-DEP cohorts (*p* = 0.52). Likewise, the comparison between the two control groups was not significant (*p* = 0.20). The burden of MGUS with an M-spike > 1 g/dL was low, with 0 in the DEP-IQ cohort, 1 in the DEP-DEU cohort, and 1 in the NON-DEP cohort.

**Table 2:** MGUS prevalence in the burn-pit exposed cohort deployed to Iraq (DEP-IQ), control cohort deployed to Germany (DEP-DEU), and control non-deployed cohort (NON-DEP).

Monoclonal gammopathy	All members (*N* = 1589)	DEP-IQ	DEP-DEU	NON-DEP	*P*-value
		(*N* = 534)	(*N* = 534)	(*N* = 521)	(Fisher’s exact test)
Serum immunofixation positive, % (*n*), 95% CI	4.7% (75), 3.7-5.9%	5.1% (27), 3.4-7.3%	3.9% (21), 2.5-5.9%	5.2% (27), 3.4-7.5%	DEP-IQ vs DEP-DEU: 0.23, DEP-IQ vs NON-DEP: 0.52, DEP-DEU vs NON-DEP: 0.20
Isotype:					
IgG, % (*n*)	68.0% (51)	74.1% (20)	76.2% (16)	55.6% (15)	
IgA, % (*n*)	18.7% (14)	11.1% (3)	19.0% (4)	25.9% (7)	
IgM, % (*n*)	8.0% (6)	7.4% (2)	0	14.8% (4)	
Lambda light chain, % (*n*)	2.7% (2)	7.4% (2)	0	0	
Biclonal, % (*n*)	2.7% (2)	0	4.8% (1)	3.7% (1)	
**OLD** light chain only ratio abnormal, % (*n*), 95% CI	1.1% (18), 0.7-1.8%	1.7% (9), 0.8-3.2%	1.5% (8), 0.7-2.9%	0.2% (1), 0-1.1%	DEP-IQ vs DEP-DEU: 0.50, DEP-IQ vs NON-DEP: 0.01, DEP-DEU vs NON-DEP: 0.02
**NEW** light chain only ratio abnormal, % (*n*)*	0.3% (5)	0.6% (3)	0.4% (2)	0	DEP-IQ vs DEP-DEU: 1, DEP-IQ vs NON-DEP: 0.25, DEP-DEU vs NON-DEP: 0.5
**OLD** serum immunofixation positive or Serum Free Light Chain (sFLC) ratio abnormal, % (*n*), 95% CI	5.9% (93), 4.7-7.1%	6.7% (36), 4.8-9.2%	5.4% (29), 3.7-7.7%	5.4% (28), 3.6-7.7%	DEP-IQ vs DEP-DEU: 0.22, DEP-IQ vs NON-DEP: 0.21, DEP-DEU vs NON-DEP: 0.54
**NEW** serum immunofixation positive or sFLC ratio abnormal, % (*n*)*	5.0% (80)	5.6% (30)	4.3% (23)	5.2% (27)	DEP-IQ vs DEP-DEU: 0.4, DEP-IQ vs NON-DEP: 0.79, DEP-DEU vs. NON-DEP: 0.56

Legacy light-chain MGUS definition yields a statistically significant difference by cohort, whereas this difference is no longer observed when the revised reference intervals are applied. *95% CI were not estimated for cells with counts less than 6 due to sparse data.

The overall prevalence of LC-MGUS, defined by conventional sFLC ratio criteria, was 1.1% (95% CI, 0.7–1.8 per 100 persons). Rates were low across all groups and did not differ significantly between the DEP-IQ and DEP-DEU cohorts (*p* = 0.50). However, the NON-DEP cohort demonstrated a significantly lower prevalence than both DEP-IQ (*p* = 0.01) and DEP-DEU (*p* = 0.02) cohorts. When revised, age-stratified reference intervals were applied based on the iStopMM proposed new reference intervals, the prevalence of LC-MGUS decreased to 0.3%, and the combined prevalence of MGUS or LC-MGUS was 5.0%, demonstrating attenuation of LC-MGUS classification with the revised definition. Isotype distribution among immunofixation-positive cases was predominantly IgG (68.0%), followed by IgA (18.7%) and IgM (8.0%), with few lambda light-chain-only or biclonal cases.

We assessed the prevalence of MGUS or revised LC-MGUS among the entire study population (*n* = 1589), as shown in Table 3. The prevalence of MGUS or revised LC-MGUS was 40/927 (4.3%) among ADSMs who had serum sampled between ages 42 (the youngest age at sampling in all three cohorts) and 49, and was 40/662 (6%) among those 50 years of age and older (*p* = 0.1206) at the time of serum sampling. The prevalence did not differ by sex, with a prevalence of 66/1,415 (4.7%) among males and 14/174 (8%) among females (*p* = 0.0542). The prevalence did differ by race, with Black ADSMs having the highest prevalence at 31/298 (10.4%), followed by Hispanic ADSMs at 6/118 (5.1%), White ADSMs at 40/1,033 (3.9%), and 3/140 (2.1%) for other/unknown race (*p* < 0.0001).

**Table 3:** Prevalence of MGUS or revised LC-MGUS by age, sex, and race.

	*N* total	MGUS + revised LC-MGUS	*p*-value
Age at sampling			0.1206
42–49	927	40 (4.3)	
50+	662	40 (6.0)	
Sex			0.0542
Male	1415	66 (4.7)	
Female	174	14 (8.0)	
Race			<0.0001
White	1033	40 (3.9)	
Black	298	31 (10.4)	
Hispanic	118	6 (5.1)	
Other/Unknown	140	3 (2.1)	

Prevalence of monoclonal gammopathy is highest in Black ADSMs.

### Comparison of deployed and non-deployed service members

For clarity, deployed service members were pooled into a single group (DEP-IQ and DEP-DEU combined; *n* = 1068) and compared with non-deployed service members (*n* = 521). MGUS was detected in 48/1068 (4.5%) of the deployed group and 27/521 (5.2%) of the non-deployed group (*p* = 0.31) (Table 4).

**Table 4:** MGUS prevalence in deployed (DEP-IQ, DEP-DEU) vs. non-deployed ADSMs.

Monoclonal Gammopathy	Deployed	Non-deployed (NON-DEP)	*P*-value
	(DEP-IQ & DEP-DEU)	(*N* = 521)	(Fisher’s exact test)
	(*N* = 1068)		
Serum immunofixation positive, % (*n*), 95% CI	4.5% (48), 3.3-5.9%	5.2% (27), 3.4-7.5%	0.31
**OLD** light chain only ratio abnormal, % (*n*), 95% CI	1.6% (17), 0.9-2.5%	0.2% (1), 0-1.1%	0.007
**NEW** light chain only ratio abnormal, % (*n*)*	0.5% (5)	0% (0)	0.25
**OLD** serum immunofixation positive or sFLC, % (*n*), 95% CI	6.1% (65), 4.7-7.7%	5.4% (28), 3.6-7.7%	0.33
**NEW** serum immunofixation positive or sFLC abnormal, % (*n*)*	5.1% (53)	5.2% (27)	0.56

Legacy light-chain MGUS definition yields a statistically significant difference between deployed and non-deployed groups, whereas this difference is no longer observed when the revised reference intervals are applied. *95% CI were not estimated for cells with counts less than 6 due to sparse data.

LC-MGUS, as defined by the conventional sFLC ratio criteria, was significantly more prevalent among deployed service members, found in 17/1068 (1.6%), compared with non-deployed service members, found in 1/521 (0.2%) (*p* = 0.007). When the revised definition of LC-MGUS was applied, 5/1068 (0.5%) met criteria in the deployed group, and 0/521 met criteria in the non-deployed group (*p* = 0.25), and therefore deployment was no longer significantly associated with LC-MGUS.

The combined prevalence of MGUS and conventionally defined LC-MGUS was numerically higher among deployed service members, occurring in 65/1068 (6.1%), than non-deployed service members, occurring in 28/521 (5.4%), although this difference did not reach statistical significance (*p* = 0.33). The combined prevalence of MGUS and the revised definition of LC-MGUS was similar between deployed service members, occurring in 53/1068 (5.1%), and non-deployed service members, occurring in 27/521 (5.2%) (*p* = 0.56).

Among the 1068 ADSMs in the DEP-IQ and DEP-DEU cohorts, the prevalence of combined MGUS and revised LC-MGUS was not affected by deployment duration, number of deployments, or deployment occupation (Table 5).

**Table 5:** MGUS prevalence by deployment characteristics.

Deployment Characteristics	*N* total	mgus+revised LC-MGUS	*p*-value
# days deployed			0.216
0	522	27 (5.2)	
6 months - 1 year	622	37 (5.9)	
> 1 year	446	16 (3.6)	
# Deployments			0.3923
0	1056	50 (4.7)	
1	278	13 (4.7)	
2	165	13 (7.9)	
3+	91	4 (4.4)	
Occupation			0.6739
Comm/intel	355	21 (5.9)	
Repair/eng	434	21 (4.8)	
Infantry/artillery/combat eng	246	13 (5.3)	
Healthcare	156	4 (2.6)	
Pilot/aircrew	72	3 (4.2)	
Motor transport	67	2 (3.0)	
Other	260	16 (6.2)	

MGUS prevalence stratified by deployment characteristics.

### Prevalence of MGUS and LC-MGUS by exposure to burn pits

To evaluate whether burn pit exposure was associated with an increased prevalence of monoclonal gammopathy, service members with documented burn pit exposure (*n* = 534) were compared with all service members without burn-pit exposure, including both non–burn pit exposed and non-deployed personnel (*n* = 1055) (Table 6).

**Table 6:** Prevalence of MGUS and LC-MGUS by burn pit exposure status.

Monoclonal gammopathy	Burn pit exposed (DEP-IQ)	Non-burn pit exposed (DEP-DEU & NON-DEP)	*P*-value
	(*N* = 534)	(*N* = 1055)	(Fisher’s exact test)
Serum immunofixation positive, % (*n*), 95% CI	5.1% (27), 3.4-7.3%	4.5% (48), 3.4-6.0%	Burn pit Exposed vs Non-Burn pit Exposed: 0.37
**OLD** light chain only ratio abnormal, % (*n*), 95% CI	1.7% (9), 0.8-3.2%	0.9% (9), 0.4-1.6%	Burn pit Exposed vs Non-Burn pit Exposed: 0.11
**NEW** light chain only ratio abnormal, % (*n*)*	0.6% (3)	0.2% (2)	Burn pit Exposed vs Non-Burn pit Exposed: 0.25
**OLD** serum immunofixation positive or sFLC, % (n), 95% CI	6.7% (36), 4.8-9.2%	5.4% (57), 0.4-6.9%	Burn pit Exposed vs Non-Burn pit Exposed: 0.17
**NEW** serum immunofixation positive or sFLC ratio abnormal, % (*n*)*	5.6% (30)	4.9% (52)	Burn pit Exposed vs Non-Burn pit Exposed: 0.79

MGUS and LC-MGUS prevalence using both legacy and revised reference intervals among burn pit–exposed and unexposed personnel. *95% CI were not estimated for cells with counts less than 6 due to sparse data.

Using serum immunofixation to define MGUS, prevalence was similar between burn pit-exposed and non-burn pit service members (5.1% vs. 4.5%, respectively; Fisher’s exact test, *p* = 0.37). When applying the revised iStopMM-based serum free light-chain reference ranges, LC-MGUS was uncommon in both groups and did not differ significantly by burn pit exposure status (0.6% vs. 0.2%; *p* = 0.25). Likewise, the combined prevalence of MGUS or LC-MGUS using revised criteria was not significantly different between burn-pit–exposed and non–burn-pit service members (5.6% vs. 4.9%; *p* = 0.79).

For comparison, analyses using conventional serum free light-chain reference intervals demonstrated a higher frequency of light chain-only abnormalities overall, with LC-MGUS identified in 1.7% of burn pit-exposed service members and 0.9% of non-burn pit service members; however, this difference was not statistically significant (*p* = 0.11). Similarly, the combined prevalence of MGUS or LC-MGUS using conventional definitions did not differ significantly between burn pit-exposed and non-burn pit groups (6.7% vs. 5.4%; *p* = 0.17).

Collectively, these findings indicate that, under both conventional and revised LC-MGUS definitions, burn pit exposure was not associated with an increased prevalence of MGUS or LC-MGUS. The absence of an association using revised reference ranges further supports the conclusion that burn pit exposure does not confer excess hematologic risk beyond that associated with military service itself.

## Discussion

In this large retrospective cohort of U.S. ADSMs, we observed a combined MGUS or LC-MGUS prevalence of 5.9%, a rate that is strikingly elevated given the relatively young age of the study population. Serum samples were obtained approximately a decade after deployment, corresponding to a median age of approximately 47 years at assessment. In the general population, MGUS prevalence at this age is well under 1%, as demonstrated by NHANES data reporting a prevalence of 0.88% among individuals aged 40–49 years [23]. Comparison with other large population-based screening efforts further underscores this observation. The Icelandic iStopMM study, which screened more than 75,000 individuals aged ≥40 years, reported an MGUS prevalence of 2.3% among individuals aged 40–59 years [24], which is substantially lower than the prevalence detected among ADSMs. Although prevalence was higher in black ADSMs than white ADSMs, both races had a higher prevalence than the breakdown by race from NHANES, which revealed a prevalence of 3.26% in Blacks and 0.53% in Whites ages 40–49 [23]. However, the iStopMM and NHANES studies may have their own inherent biases.

There are several explanations for the high observed prevalence of MGUS in our ADSM population, including selection bias related to the eligibility criteria for this study, survivorship bias, and the study population, which included older ADSMs who were mostly men with a higher percentage of Blacks than in the general U.S. population. It is also possible that factors beyond age, sex, and race may contribute to plasma cell dysregulation in military populations. For example, volunteerism in the current All-Volunteer Force may not necessarily be reflected in the demographics of the ADSMs included in this study and may have other risk factors that influence MGUS prevalence.

Despite the elevated overall prevalence, MGUS was not associated with deployment location or reported burn pit exposure. Service members deployed to Iraq with burn pit exposure demonstrated MGUS prevalence rates similar to those observed in deployed Germany controls and non-deployed ADSMs. These results align with prior epidemiologic studies that have not consistently demonstrated increased hematologic malignancy risk associated with burn pit exposure [15], despite strong biological plausibility based on the composition of burn pit emissions. The null association may be attributable to exposure measurement error rather than a true absence of biologic effect, as burn pit exposure was assessed by self-report and therefore subject to recall bias. Collectively, our findings suggest that burn pit exposure alone is unlikely to be a dominant driver of plasma cell abnormalities in service members.

Importantly, our study highlights the methodological sensitivity of LC-MGUS classification to sFLC reference intervals. Using conventional sFLC thresholds, deployment was associated with an increased prevalence of LC-MGUS; however, this association was attenuated and no longer statistically significant when revised, age-stratified reference ranges derived from the iStopMM study were applied, indicating that the observed relationship is sensitive to the choice of diagnostic thresholds. These findings highlight the limitations of conventional sFLC reference intervals, which have been shown to systematically over diagnosis light-chain abnormalities.

More broadly, these methodological considerations have implications that extend beyond this study and are relevant to plasma cell disorder research in general. The marked attenuation of LC-MGUS prevalence and exposure associations following application of revised reference ranges suggests that prior reports of elevated LC-MGUS prevalence in specific populations may, at least in part, reflect misclassification driven by outdated diagnostic criteria rather than true biological differences. While signals observed using conventional definitions may still reflect underlying biological phenomena, such as transient immune activation or altered light-chain dynamics, longitudinal studies incorporating repeated biomarker assessment, genomic characterization, and refined exposure data will be essential to distinguish transient perturbations from true clonal plasma cell disorders.

Beyond the specific question of burn pit exposure, our findings raise broader considerations regarding the cumulative environmental and occupational exposures inherent to military service. ADSMs may encounter a range of potentially immunotoxic exposures, including combustion products, industrial chemicals, fuels, particulate matter, and chronic inflammatory stressors, which may collectively influence immune regulation and plasma cell biology [25, 26]. These exposures may not be captured by deployment location or self-reported burn pit exposure alone, underscoring the complexity of exposure assessment in military epidemiology.

This study has several strengths, including a large, well-matched cohort, long-term post-deployment follow-up, and rigorous laboratory assessment incorporating updated diagnostic criteria. However, limitations warrant consideration. Burn pit exposure was self-reported and may be subject to misclassification. Serum samples were collected at a single time point, precluding assessment of clonal persistence or progression risk. Because of the young age of ADSMs included in this study, as well as strict retention standards for military service, all ADSMs were presumed to have normal renal function, which could result in potential confounding during assessments of LC-MGUS using the revised classification. Additionally, although MGUS prevalence was elevated when compared to certain populations, the clinical significance of these findings, particularly with respect to progression to MM or related disorders, remains unknown, particularly since the vast majority of MGUS detected had an M-spike less than 1 g/dL.

In conclusion, MGUS prevalence was unexpectedly high among post-9/11 ADSMs and was not associated with burn pit exposure. MGUS or revised LC-MGUS was identified in 5.6% of DEP-IQ, 4.3% of DEP-DEU, and 5.2% of NON-DEP, yielding an overall prevalence of 5%, with markedly higher rates in Black compared with White ADSMs (10.4% vs. 3.9%). These findings suggest that military service may involve broader or cumulative risk factors for plasma cell dysregulation beyond specific deployment-related exposures. Longitudinal studies with serial biospecimen collection, refined exposure assessment, and integration of genomic and immunologic data will be essential to define progression risk and inform evidence-based screening [27] and surveillance strategies and risk mitigation for military and veteran populations.

### Disclaimer

The contents of this publication are the sole responsibility of the author(s) and do not necessarily reflect the views, opinions, or policies of Uniformed Services University of the Health Sciences (USUHS), The Henry M. Jackson Foundation for the Advancement of Military Medicine, Inc., the Department of Defense (DoD), or the Departments of the Army, Navy, or Air Force. Mention of trade names, commercial products, or organizations does not imply endorsement by the U.S. Government. The content is solely the responsibility of the authors and does not necessarily represent the official views of the NIH.

## Supplementary information

Reproducibility checklist

## Data Availability

All data are available by request to the Armed Forces Health Surveillance Division.
